# Hybridising inorganic materials with fluorescent BOPHY dyes: A structural and optical comparative study

**DOI:** 10.3389/fchem.2022.921112

**Published:** 2022-06-28

**Authors:** Umar Sani, Omar M. Alatawi, Nuha M. Halawani, Jamie A. Gould, Julian G. Knight, Fabio Cucinotta

**Affiliations:** ^1^ School of Natural and Environmental Sciences, Newcastle University, Newcastle upon Tyne, United Kingdom; ^2^ Department of Chemistry, Faculty of Applied Science, Umm Al-Qura University, Makkah, Saudi Arabia

**Keywords:** mesoporous silica, light-harvesting, hybrid materials, luminescence anisotropy, sol-gel syntesis, BOPHY dyes

## Abstract

This study presents the design and characterization of new monochromatic light-harvesting systems based on inorganic porous materials hybridized with organic dye molecules within their structure. A new fluorescent BOPHY dye was prepared, characterized optically and used as both reference and synthetic precursor for two alkoxysilane derivatives that were incorporated separately within a silica structure. The dyes, one bearing one alkoxysilane group and the other one two, were co-condensed with tetraethyl orthosilicate to form a hybrid organo-silica framework, where they are found at specific locations. The structure of the new materials was analysed by powder XRD and TEM, which confirmed the presence of the hexagonal pore arrangement typical of mesoporous MCM-41 silica particles. The steady-state and time-resolved analysis showed that the particles where the dyes are most dispersed within the framework retain the highest fluorescence quantum yield, up to 0.63, in the green-yellow region of the visible spectrum. On the other hand, increasing the content of BOPHY units in the solid matrix seem to favour non-radiative deactivation pathways and aggregation phenomena, which lower the efficiency of light emission. The materials also exhibit interesting properties, such as a dual excited-state decay and fluorescence anisotropy. The short fluorescence lifetime, about 2 ns, matches the typical singlet lifetime of BOPHY dyes, whereas the long component, up to 20 ns, is attributed to delayed fluorescence, which could take place via charge recombination. Optical anisotropy experiments revealed that all materials show polarised light emission to a significant extent and, for most samples, it was also possible to determine a polarisation transfer decay trace, from 400 to 800 ps This is ascribed to the occurrence of energy migration between neighbouring dye units within the silica structure.

## 1 Introduction

Research on artificial light-harvesting antennae has made considerable progress with molecular systems and the mechanism of light collection and transfer has been well established for assemblies operating on a linear chain mode. (Calver et al., 2016; La Mazza et al., 2016; Kownacki et al., 2019). Important advancements have also been achieved with the realisation of materials in which a large number of chromophores are assembled at certain levels of organisation (Zeng et al., 2017; Rocard et al., 2018); the challenge, though, still lies in the generation of antenna materials capable of activating multiple excitonic sites at a high rate and in a localised 3-dimensional space. Such materials are of primary need for applications in photocatalysis, where the bottlenecks are represented by multi-electronic reactions (Harriman, 2013; Armaroli and Balzani, 2016).

An appealing class of materials for the above purpose is that of inorganic porous frameworks where the structure can be hybridised with organic components. Periodic mesoporous organosilica (PMO) is one of the most studied types and, since the first developments in 1999 (Asefa et al., 1999; Inagaki et al., 1999; Melde et al., 1999), they have witnessed a vast growth, with gradual emergence of more complex structured architectures (Cornelius et al., 2005; van der Voort et al., 2013). From small organic groups like benzene and biphenyl, the range of dopants has been extended to chromophores such as pyrene and porphyrines (Jeong et al., 2010; Mizoshita et al., 2010), to include even transition metal complexes like ruthenium tris-bipyridine (Takeda et al., 2016). The latter showed potential as an artificial photosynthetic system when coupled with water oxidation catalysts. On the other hand, the major disadvantages of PMO materials are that most of the silicate precursors are not commercially available and so must be synthesised specifically; also, any technological application will struggle to progress on large scales unless cheaper materials are used rather than rare metals.

We recently focused on the realisation of small hybrid inorganic materials using BODIPY and BOPHY fluorophores as dopants (Cucinotta et al., 2018). Typically, the core structure of such dyes is relatively easy to modify through the introduction of specific functional groups at a variety of different positions, allowing fine changes to absorption and emission wavelengths. This opens the way to several applications as biological probes, fluorescent sensors and markers, light-harvesting agents and light emitters in optoelectronic devices (Lv et al., 2018; Sola-Llano et al., 2019; Bismillah and Aprahamian, 2021).

**FIGURE 1:**
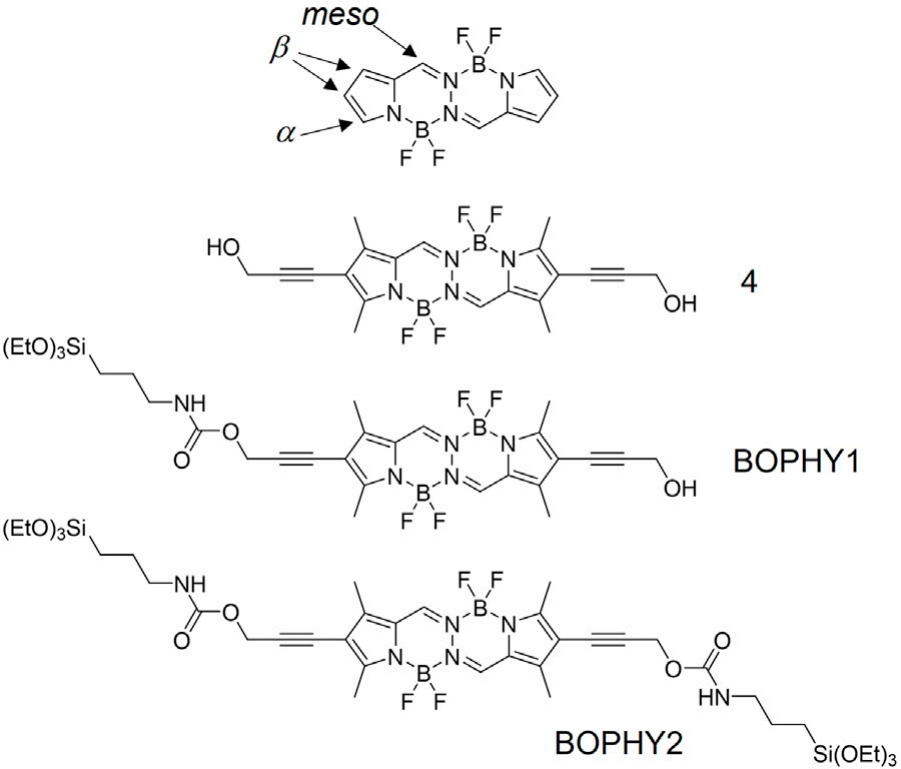
Chemical structures of the dyes presented in this study, in comparison with a core BOPHY (top structure).

BOPHY dyes are particularly interesting owing to their extended conjugated structure. They comprise a tetracyclic (5,6,6,5) ring system in which each of the central two six-membered BF_2_ chelates is π-extended by fusion of a pyrrole ring (see Figure 1, top structure). (Boodts et al., 2018) This gives BOPHYs a very stable π-conjugated core structure and desirable optical properties, such as high molar extinction coefficients (80,000–100,000 M^−1^ cm^−1^) and quantum yields (around 70%–80%). These dyes can easily be prepared via a particularly straightforward two-step one-pot synthesis in water or common organic solvents. Although the synthesis of BOPHYs resembles that of BODIPYs, it is typically higher yielding, more amenable to scale-up and suffers from an improved impurity profile. Also, fine-tuning of the optical properties of both BOPHYs and BODIPYs can be obtained with relative simplicity through the extension of the π-conjugated system, using either commercially available or synthesised pyrroles. (Li et al., 2016). Owing to such advantageous properties, both types of dyes have been further studied as part of supramolecular architectures, where covalent attachment or self-assembly was pursued to achieve multi-dye systems with even broader applications than those of a single dye. (Chen et al., 2013; Jiang et al., 2015; Rhoda et al., 2015). A recent work by Mirloup et al (2015) showcased their application in bulk heterojunction solar cells, which exhibited broad absorption in the whole visible spectrum. However, as all fluorophores, they have limited photochemical stability, a drawback that has prompted research into effective strategies for extended use in devices, one of which involves the incorporation into inorganic hosts.

In this study, we present new light-harvesting antennae, where BOPHY dyes are hybridised within a porous inorganic framework, yielding new PMO materials. The structural and optical properties will also be discussed and compared with those of a precursor dye, to evaluate whether the new materials possess improved and also emerging optical properties. Two BOPHY derivatives have been used, as shown in Figure 1. BOPHY1 bears an alkoxysilane side group, whereas BOPHY2 possesses two of them. Both were synthesised by means of a simple and relatively easy route, which represents a convenient advantage. The use of each dye was expected to have a different impact on the structure of the PMO materials built from them and also on their luminescence behaviour.

## 2 Materials and methods

All air- and water-sensitive experiments were performed under a nitrogen atmosphere by using standard vacuum-line techniques. All chemicals were obtained commercially and used without further purification. The synthetic precursors of 4 were synthesised according to procedures reported in the Supplementary Material S1–S13. Thin-layer chromatography (TLC) was carried out on silica plates (silica gel 60 F254, Merck 5554) and visualized by UV lamp (254 nm). NMR spectra were recorded with either Bruker AVANCE 300 MHz or JEOL 400 MHz spectrometers operating at 25°C. Transmission electron microscopy images were taken on a 100 kV CM100 TEM (FEI). Powder X-ray diffraction (powder XRD) was performed utilising a PANalytical X'Pert Pro MPD, powered by a Philips PW3040/60 X-ray generator fitted with an X'Celerator detector. Diffraction data were acquired by exposing powder samples to Cu-Kα X-ray radiation, which has a characteristic wavelength (λ) of 1.5418 Å. X-rays were generated from a Cu anode supplied with 40 kV and a current of 40 mA. Data sets were collected over a range of 1°–20° 2θ with a step size of 0.0334° 2θ and nominal time per step of 1 s, using the scanning X’Celerator detector and a Ni Kβ filter in the diffracted beam path. The optics set up for the instrument are as follows; programmable divergence slit with a fixed width of 1/32°, an incident anti-scatter slit of 1/16°, a beam mask of 20 mm and incident/diffracted Soller slits of 0.04 radians.

### 2.1 Synthesis of 4: 3,3′-(5,5,12,12-tetrafluoro-1,3,8,10-tetramethyl-5*H*,12*H*-5l4,6l4,12l4,13l4-pyrrolo[1,2-d]pyrrolo[1′,2′:4,5][1,2,4,3]triazaborinino[2,1-a][1,2,4,3]triazaborinine-2,9-diyl)bis(prop-2-yn-1-ol)

5,5,12,12-tetrafluoro-2,9-diiodo-1,3,8,10-tetramethyl-5*H*,12*H*-5l4,6l4,12l4,13l4-pyrrolo [1,2-d]pyrrolo [1′,2′:4,5][1,2,4,3]triazaborinino [2,1-a][1,2,4,3]triazaborinine 3 (van der Voort et al., 2013) (0.200 g, 0.338 mmol, 1 eq) CuI (0.039 g, 0.206 mmol, 0.61 eq) and [1,1′bis (diphenylphosphino)ferrocene]dichloropalladium (II), complex with dichloromethane [Pd (dppf)Cl_2_]CH_2_Cl_2_ (0.080 g, 0.101 mmol, 0.3 eq) were dissolved in THF (10 ml). Triethylamine (3 ml) was added, followed by propargyl alcohol (0.093 g, 1.66 mmol, 4.9 eq), the reaction mixture was heated at 75°C for 40 h. After that, the reaction mixture was allowed to cool to room temperature and then washed with water (2 × 15 ml) then 1M HCl (2 × 20 ml) and extracted with CH_2_Cl_2_ (2 × 30 ml). The combined organic layers were dried over MgSO_4_ and filtered. The organic solvent was removed under reduced pressure to yield a solid which was purified by column chromatography (petroleum ether: acetone 2:1) to give the title compound 4 as an orange solid (0.100 g, 66%), R_*f*_ = 0.25 (petroleum ether: acetone 2:1). M. p. 268–273 °C. ^1^H NMR (300 MHz, CDCl_3_) δ 7.96 (s, 2H), 4.55 (s, 4H), 2.55 (s, 6H), 2.37 (s, 6H) (Mizoshita et al., 2010).C NMR (101 MHz, CDCl_3_) δ 153.74, 142.77, 135.47, 122.24, 113.29, 93.82, 51.76, 13.10, 10.52.^11^B NMR (96 MHz, CDCl_3_) δ 0.62 (t, *J*_*B-F*_ = 28.1 Hz). ^19^F NMR (282 MHz, CDCl_3_) δ δ -142.50–-143.05 (m). IR (neat): νmax/cm^−1^: 3304, 2,921, 2,177, 1581, 1479, 1382, 1302, 1238, 991, 857, 716, 553, 491. Found: [M - F]^+^; 445.1641, C_20_H_20_B_2_F_4_N_4_O_2_: requires [M - F]^+^ 445.1637. See spectra in the Supplementary Material S14–S17.

### 2.2 Synthesis of BOPHY1: 3-(5,5,12,12-tetrafluoro-9-(3-hydroxyprop-1-yn-1-yl)-1,3,8,10-tetramethyl-5H,12H-5l4,6l4,12l4,13l4-pyrrolo[1,2-d]pyrrolo[1′,2′:4,5][1,2,4,3]triazaborinino[2,1-a][1,2,4,3]triazaborinin-2-yl)prop-2-yn-1-yl (3-(triethoxysilyl)propyl)carbamate

4 (0.043 g, 0.096 mmol, 1 eq) was dissolved in dry THF (10 ml). 3-(Triethoxysilyl)propyl isocyanate (0.029 g, 0.115 mmol, 1.2 eq) was added, followed by triethylamine (0.002 g, 0.019 mmol, 0.2 eq), the reaction was heated under reflux for 42 h the reaction mixture was allowed to cool to room temperature and then the organic solvent was removed under reduced pressure to yield a solid which was purified by column chromatography (petroleum ether: ethyl acetate 1:1) to give the title compound 5 as an orange gum (0.022 g, 32%), R_*f*_ = 0.5 (petroleum ether: ethyl acetate 1:1).^1^H NMR (300 MHz, CDCl_3_) δ 7.95 (s, 2H), 5.07 (s, 1H), 4.94 (s, 2H), 4.55 (s, 2H), 3.82 (q, *J* = 7.2 Hz, 6H), 3.22 (q, *J* = 6.6 Hz, 2H), 2.55 (s, 6H), 2.37 (s, 6H), 1.70–1.62 (m, 2H), 1.24 (t, *J* = 7.0 Hz, 9H), 0.70–0.58 (m, 2H). ^13^C NMR (101 MHz, CDCl_3_) δ 155.55, 153.98, 143.06, 135.50, 122.21, 113.10, 92.92, 90.41, 58.39, 53.19, 43.53, 23.24, 18.29, 13.11, 10.53, 7.65.^11^B NMR (96 MHz, CDCl_3_) δ 1.13–-0.39 (m). ^19^F NMR (282 MHz, CDCl_3_) δ -142.50–-143.05 (m). See spectra in the Supplementary Material S18–S21.

### 2.3 Synthesis of BOPHY2

(5,5,12,12-tetrafluoro-1,3,8,10-tetramethyl-5*H*,12*H*-5l4,6l4,12l4,13l4-pyrrolo [1,2-*d*]pyrrolo [1′,2′:4,5][1,2,4,3]triazaborinino [2,1-a][1,2,4,3]triazaborinine-2,9-diyl)bis (prop-2-yne-3,1-diyl) bis((3-(triethoxysilyl)propyl)carbamate). 4 (0.030, 0.067 mmol, 1 eq) was dissolved in dry THF (10 ml). 3-(Triethoxysilyl)propyl isocyanate was added (0.200 g, 0.807 mmol, 12 eq) followed by triethylamine (0.041 g, 0.403 mmol, 6 eq), the reaction was refluxed for 42 h. The reaction mixture was allowed to cool to room temperature and then the organic solvent was removed under reduced pressure to yield a solid which was purified by column chromatography (petroleum ether: ethyl acetate 1:1) to give the title compound 6 as an orange solid (0.038 g, 28%), R_*f*_ = 0.7 (petroleum ether: ethyl acetate 1:1). ^1^H NMR (300 MHz, CDCl_3_) δ 7.95 (s, 2H), 5.07 (br s, 2H), 4.94 (s, 4H), 3.82 (q, *J* = 7.0 Hz, 12H), 3.21 (q, *J* = 6.6 Hz, 4H), 2.54 (s, 6H), 2.37 (s, 6H), 1.66–1.61 (m, 4H), 1.22 (t, *J* = 7.0 Hz, 18H), 0.70–0.58 (m, 4H). ^13^C NMR (101 MHz, CDCl_3_) δ 155.55, 153.97, 143.05, 135.50, 122.21, 113.11, 90.41, 58.48, 53.18, 43.53, 23.23, 18.43, 18.27, 13.10, 10.52, 7.65.^11^B NMR (128 MHz, CDCl_3_) δ 0.90–0.07 (m). ^19^F NMR (282 MHz, CDCl_3_) δ -142.74–-143.01 (m). IR (neat): ν_max_/cm^−1^: 3,317, 3,073, 2,974, 2,928, 2,885, 2,767, 2,735, 2,235, 1,699, 1,591, 1,541, 1,483, 1,441, 1,375, 1,308, 1,239, 1,205, 1,123, 1,059, 998, 863, 774. Found [M^+^ - F]; 921.4236 C_40_H_62_B_2_F_4_N_6_O_10_Si_2_: requires [M^+^ - F] 921.4219. See spectra in the Supplementary Material S22–S25.

### 2.4 Synthesis of the BOPHY1-PMOs

Cetyltrimethylammonium bromide (CTAB) was dissolved in 30 ml of water using an ultrasound bath. This was then followed by the addition of 2 M NaOH (0.13 ml, 0.26 mmol). The solution was heated to 100°C. BOPHY 1 was dissolved in a minimal amount of water (6 ml) and added dropwise concurrently with TEOS (360 µl), forming a homogeneous turbid orange mixture. The mixture was heated under reflux for 2 h, and then allowed to cool down to room temperature. The mixture was filtered under vacuum and washed with water. The filtrate was dried in a desiccator, and a pink solid product was obtained that fluoresced lightly under a UV lamp. The TEOS and BOPHY 1 were added to each reaction in the following ratios; 99:1 (69.3 mg, 0.198 mmol and 1.39 mg, 2.00 µmol), 98:2 (68.6 mg, 0.196 mmol and 2.77 mg, 4.00 µmol) and 95:5 (66.5 mg, 0.190 mmol and 6.93 mg, 10.00 µmol).

### 2.5 Synthesis of the BOPHY2-PMOs

Cetyltrimethylammonium bromide (CTAB) was dissolved in 30 ml of water using an ultrasound bath. This was then followed by the addition of 2 M NaOH (0.13 ml, 0.26 mmol). The solution was heated to 100°C. BOPHY 2 was dissolved in a minimal amount of water (6 ml) and added dropwise concurrently with TEOS (360 µl), forming a homogeneous turbid orange mixture. The mixture was heated under reflux for 2 h, and then allowed to cool down to room temperature. The mixture was filtered under vacuum and washed with water. The filtrate was dried in a desiccator, and a pink solid product was obtained that fluoresced lightly under a UV lamp. The TEOS and BOPHY 2 were added to each reaction in the following ratios; 99:1 (69.3 mg, 0.198 mmol and 1.88 mg, 2.00 µmol), 98:2 (68.6 mg, 0.196 mmol and 3.76 mg, 4.00 µmol) and 95:5 (66.5 mg, 0.190 mmol and 9.4 mg, 10.00 µmol).

### 2.6 UV–vis spectroscopy analysis

Absorption spectra were recorded using a Shimadzu UV-1800 spectrophotometer. Luminescence spectra and excited state lifetimes were measured using an Edinburgh FLS980 photoluminescence spectrometer, equipped with a 450 W Xenon arc lamp, Czerny Turner excitation and emission monochromators (1.8 nm/mm dispersion; 1800 grooves/mm), time-correlated single photon counting (TCSPC) module and a Hamamatsu R928 P photomultiplier tube (in a fan assisted TE cooled housing, operating temperature −20°C). For lifetime measurements, samples were excited with an EPL-375 (370.8 nm; 61.1 ps pulse width) and an EPL-475 (471.8 nm; 61.1 ps pulse width) picosecond pulsed diode lasers and data analysis was performed on the F980 software with numerical data reconvolution based on Marquardt-Levenberg algorithm. Luminescence quantum yields were measured using as references basic fluorescein, whereas for the dye-doped samples the respective free dyes were used as reference.

## 3 Results and discussion

### 3.1 The BOPHY diol dye

**FIGURE 2:**
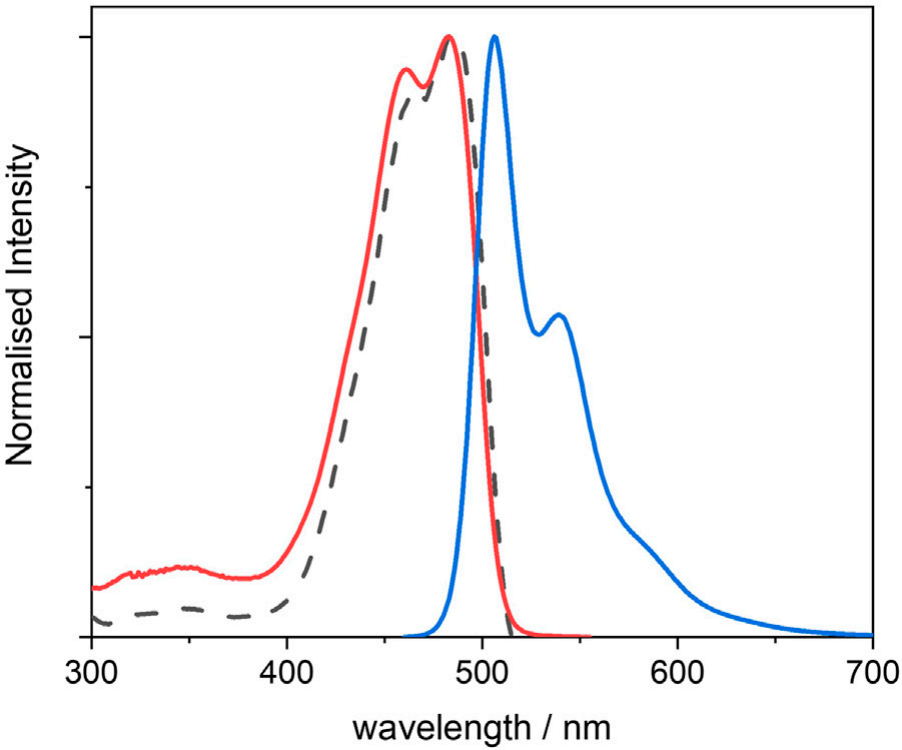
Normalised UV-visible absorption (red), excitation (dotted black) and emission (blue) spectra of 4, recorded from 10^−6^ M THF solutions; excitation was measured at λ_em_ = 540 nm, and emission was measured using λ_exc_ = 440 nm.

The BOPHY diol 4 is a new compound and it has been designed to be used as both reference dye and synthetic precursor for the other two BOPHY dyes used in this study. The synthesis of 4 was accomplished by double palladium-catalysed Sonogashira coupling of the corresponding 2,9-diiodoBOPHY (van der Voort et al., 2013) with excess propargyl alcohol (see Section 2.1). The UV-visible spectra presented here (Figure 2) are measured from a diluted solution of 4 and they are used as comparison for the PMO materials that are built using its synthetic derivatives and are discussed later.

The dye in tetrahydrofuran (THF) shows two distinct absorption peaks with λ_max_ at 467 and 487 nm, the latter with a molar extinction coefficient of 45,600 M^−1^ cm^−1^ (see also Supplementary Material S26). The absorption appears to be red-shifted as compared with that of non-functionalised BOPHY dyes (peaks at 424 and 442 nm). (Asefa et al., 1999; Inagaki et al., 1999). This is a result of the reduced optical gap due to the increased conjugation and to the electron-donating character of the four methyl substituent groups on the pyrrole rings. The structured shape of the absorption reveals the vibronic progression within the S_0_ → S_1_ electronic transition and it confirms the rigidity of the dye.

The photoluminescence spectrum also exhibits a structured profile, with peaks at 506 and 539 nm, and a small Stokes shift of 771 cm^−1^ with the lowest-energy absorption peak. This confirms that the molecule undergoes relatively small geometric changes between absorption and emission, with little energy lost through vibration at the excited state. Indeed, the fluorescence quantum yield is high (0.86). The excited state lifetime was also measured (see Supplementary Material S27) and its value of 2.8 ns is in line with typical singlet decay times from organic fluorophores.

Incorporation of propargyl alcohol substituents provides sterically unencumbered tethers for further functionalisation at the β positions. In order to incorporate the dye inside the silica framework, alkoxysilane groups were introduced at one β position only (BOPHY1) and at two β positions (BOPHY2). Chemoselective mono-alkoxysilylation was achieved by reaction of 4 with 1 molar equivalent of 3-(triethoxysilane)propyl isocyanate, to give BOPHY1. Double alkoxysilylation to give BOPHY2 was accomplished by reaction of the diol 4 with excess of the isocyanate (see Sections 2.2 and 2.3). The product obtained was used straight after chromatographic purification to prevent decomposition, since previous reports show that alkoxysilane derivatives typically undergo fast hydrolysis and/or oligomerisation.

**FIGURE 3:**
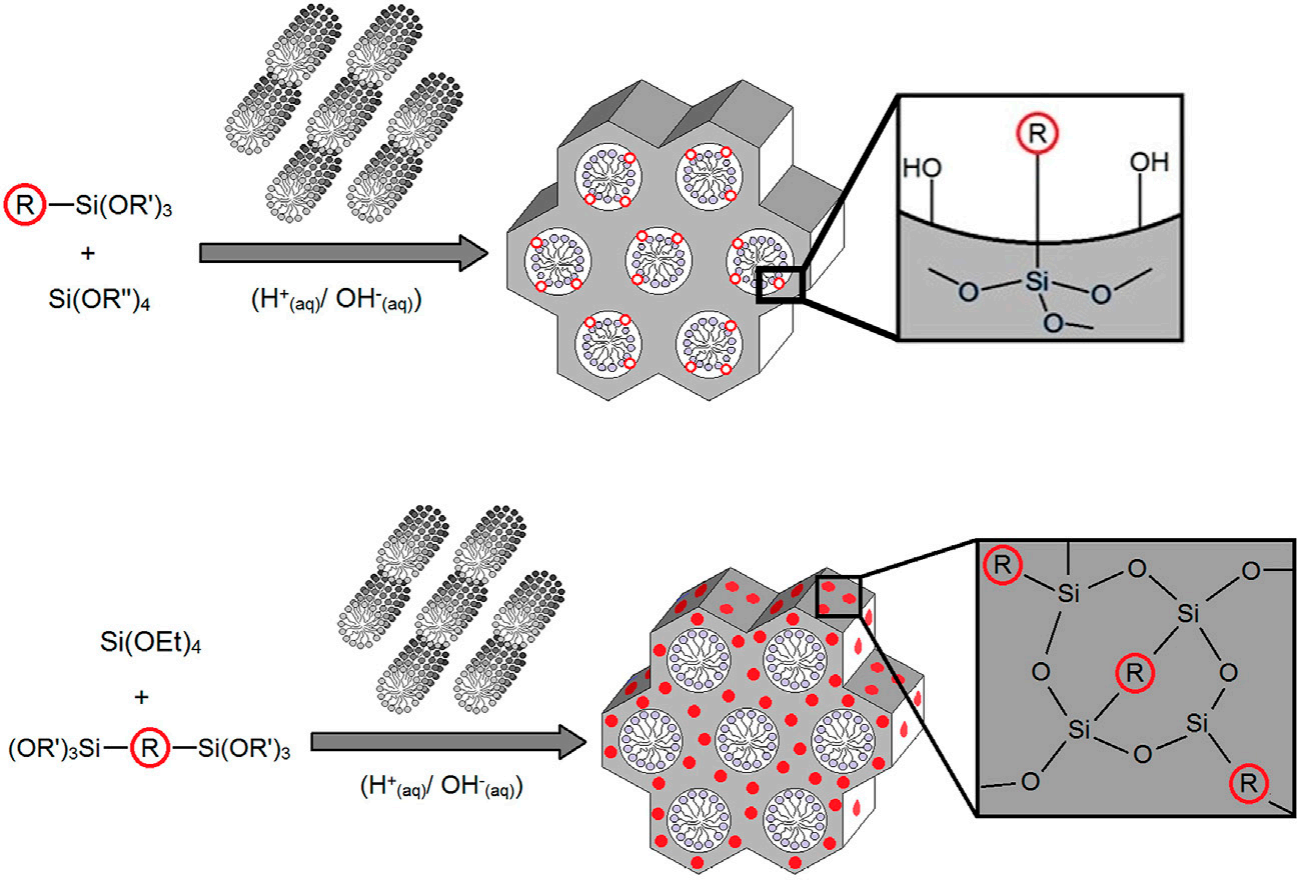
General synthetic route for the PMOs made using BOPHY1 (top) and BOPHY2 (bottom), where R = BOPHY core structure.

For the synthesis of the PMOs, we then adapted the templated synthetic procedure for mesoporous MCM-41 silica (Hoffmann et al., 2006) for the two different BOPHYs separately. In such a process, a soft template is used, which consists of CTAB micelles and acts as a structure-directing agent for the growth of the organosilica matrix (see Figure 3, above the reaction arrow). Around such template, the dyes undergo hydrolysis and poly-condensation jointly with tetraethyl orthosilicate (TEOS), although leading to different outcomes. As illustrated in Figure 3, the PMO materials made using BOPHY1 have the chromophoric units dispersed through the solid network at the silica interface with the template. Differently from its mono-silylated homologue, BOPHY2 possesses two alkoxysilane side groups and its poly-condensation with TEOS is expected to make the chromophoric core a bridging unit between the siloxane groups. This results in the dye becoming an integral part of the silica framework. In order to obtain a homogeneous dispersion of the dye, the addition into the reaction mixture was made simultaneously with TEOS and it was facilitated by pre-dispersing the dyes in the minimum amount of water with the aid of ultra-sound. Co-condensation of both dye and TEOS was then allowed to proceed under reflux conditions (see Sections 2.4 and 2.5). At the end of the PMO synthesis, the soft template is retained within the structure, as empty mesopores are not needed for the purpose of the present study and the presence of the template does not alter the optical properties of the materials.

### 3.2 PMO materials with BOPHY1

#### 3.2.1 Structural analysis

The structure and the morphology of porous silica are often disrupted with the increase in the concentration of dopants, particularly in those cases where the dyes are bulky. BOPHY dyes are relatively small, instead, and we would not expect significant disruptions upon incorporation within the silica structure. Nevertheless, to avoid heavy structural deformations, low dye concentrations were used, with relative molar ratios BOPHY1:TEOS of 1%, 2%, and 5%.

**FIGURE 4:**
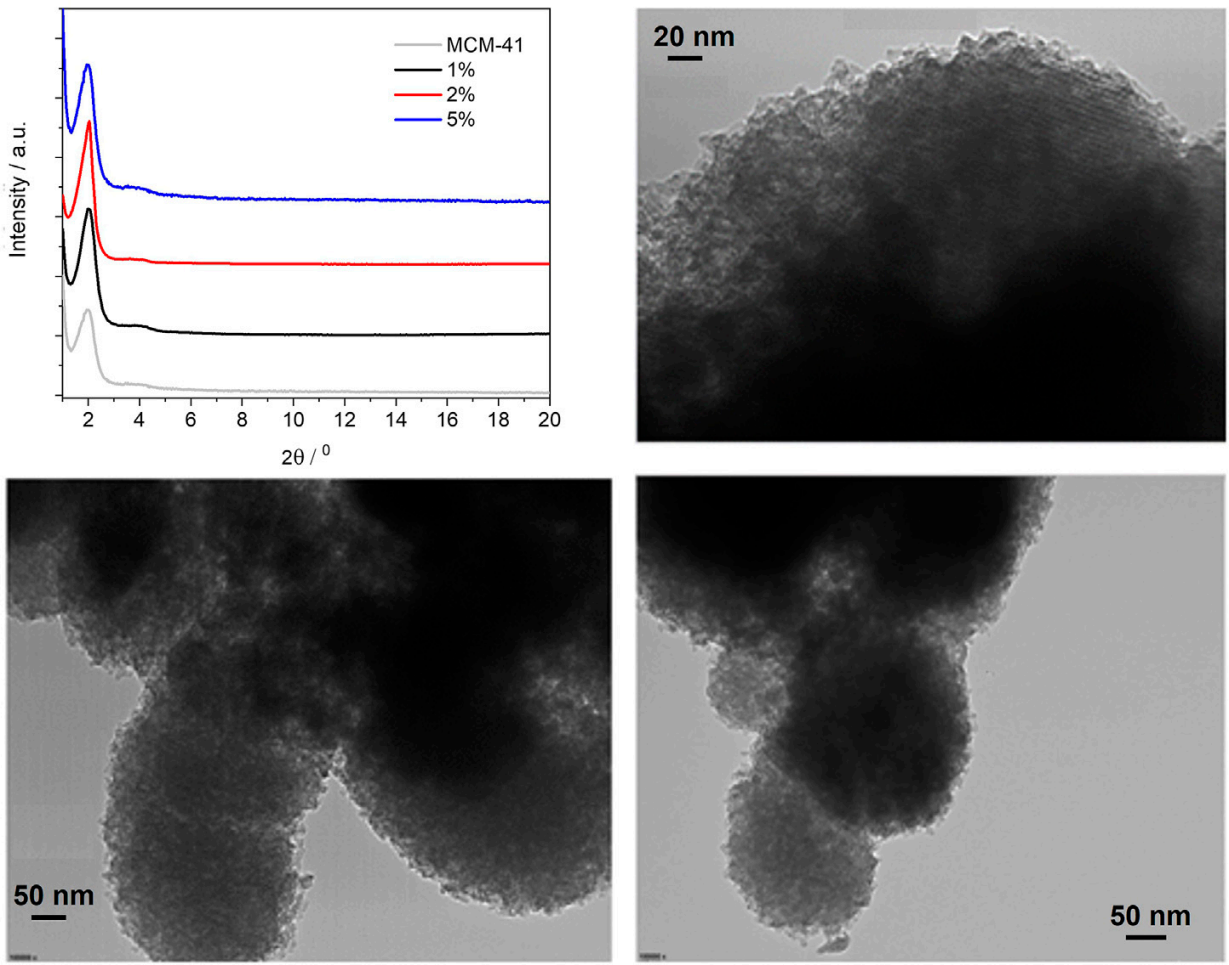
Powder X-ray diffraction patterns of the BOPHY1-PMO materials (top left); TEM images of 1% (top right), 2% (bottom left) and 5% (bottom right).

Powder XRD was then performed on the three PMO materials (see Figure 4) and revealed no changes in structure as compared with plain MCM-41 silica. In all three PMOs, the intense reflection from the (100) plane can be observed at 2θ = 1.97°, which gives a *d*-spacing of 4.48 nm. This is typically taken as the distance between the centres of adjacent pores in the hexagonal array. (Zeng et al., 2017). It is also possible to observe the (110) reflection, although broad and weak, around 3.8°, which is indicative of long-range ordering within the materials.

The TEM images confirm that the porous structure remains unaltered, with only a slight reduction of the pores diameter, from about 5 nm in the 1% material to 4 nm in the 5%. Also, it can be observed that the particles morphology does not change with dye loading and is kept near spherical.

#### 3.2.2 Photophysical properties

**FIGURE 5:**
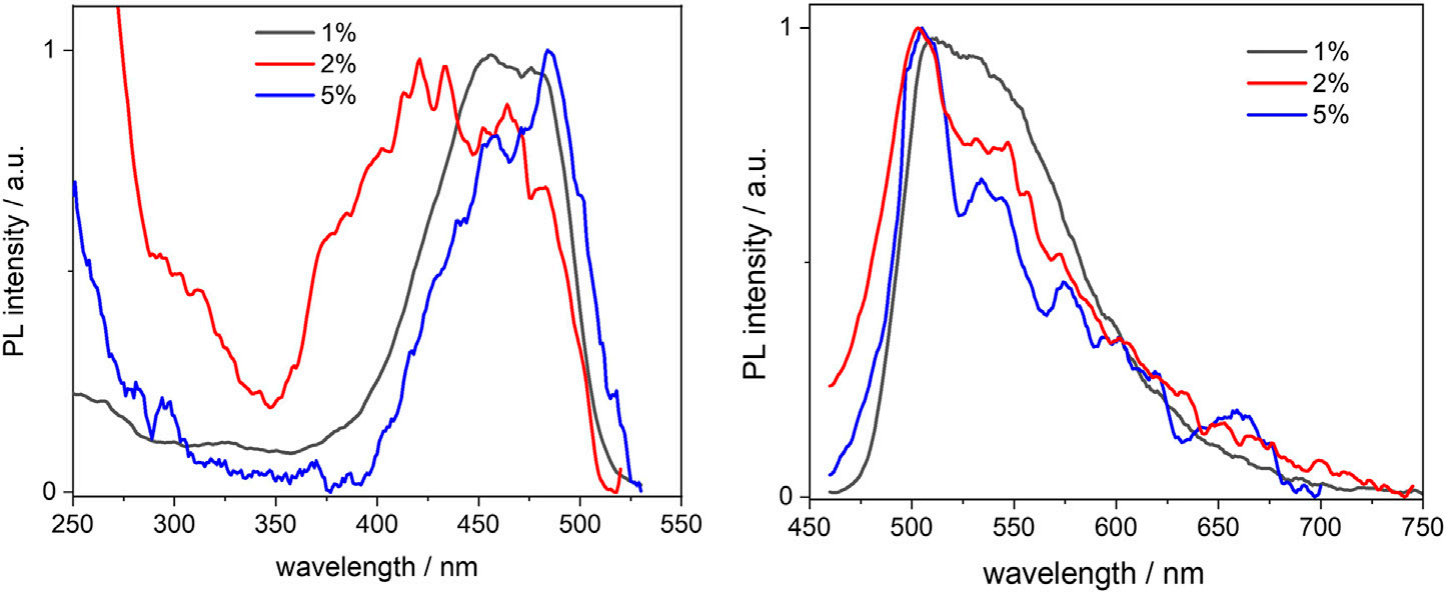
Normalised excitation (left) and emission (right) spectra of the BOPHY1-PMO samples, recorded from 0.3 mg/ml cyclohexane suspensions at λ_em_ = 540 nm and λ_exc_ = 420 nm, respectively.

The photoluminescence spectra of the three BOPHY1-PMO materials were measured from 0.3 mg/ml suspensions in cyclohexane and are shown in Figure 5. Suspensions were preferred over solid-state thin films because of the reduced light scattering from the nanoparticles. Also, cyclohexane was found an ideal solvent of choice, owing to its ability to aid the formation of fine and durable suspensions, and its inertness towards the dye units and the soft template retained in the PMO structure; in fact, CTAB is completely insoluble in cyclohexane and the dye is covalently bound and an integral part of the PMO material.

No considerable changes were observed when compared with the free complex, except for an apparent blue-shift of the excitation peaks. This is particularly noticeable in the 1% sample, where the excitation maxima lie at 456 and 478 nm, about 10 nm blue-shifted in comparison with the reference dye. As the loading increases, the excitation band broadens and this might be due to the closer proximity between single dye units, which causes aggregation. Such an effect is not as much pronounced in the emission spectra, which are very similar and match with that of the free dye, apart from the scattering effects that are visible in the noisy pattern. The fluorescence quantum yields, though, seem to indicate that the molecules do engage in some intermolecular processes of non-radiative deactivation, such as quenching, when the loading increases: the yields are reduced by a factor of nearly 4 from a value of 0.53 at 1% loading (see also Table 1). Vu and co-workers have previously reported a similar trend when studying the aggregation of BODIPY dyes in rigid matrices. (Vu et al., 2013). Aggregation between single units of dye can easily produce dimers or excimers, with partially overlapped spectra and shorter fluorescence lifetimes.

**TABLE 1:** Excited-state lifetimes, fluorescence quantum yields, radiative rate constants (k_r_) and non-radiative rate constants (k_nr_) of the BOPHY1-PMOs.

Sample	Decay traces [ns] (relative weight)^a^	Average τ_PL_ [ns]	Φ_PL_	k_r_ [s^−1^]	k_nr_ [s^−1^]
**4**	2.8	2.8	0.86	3.1 × 10^8^	5.2 × 10^7^
**1%**	2.6 (73%)–11.3 (27%)	4.9	0.53	1.1 × 10^8^	9.6 × 10^7^
**2%**	2.7 (76%)–12.6 (24%)	5.1	0.14	2.7 × 10^7^	1.7 × 10^8^
**5%**	2.3 (77%)–10.5 (23%)	4.2	0.11	4.8 × 10^6^	2.1 × 10^8^

a Recorded at 540 nm for all samples, using a 475 nm laser excitation.

**FIGURE 6:**
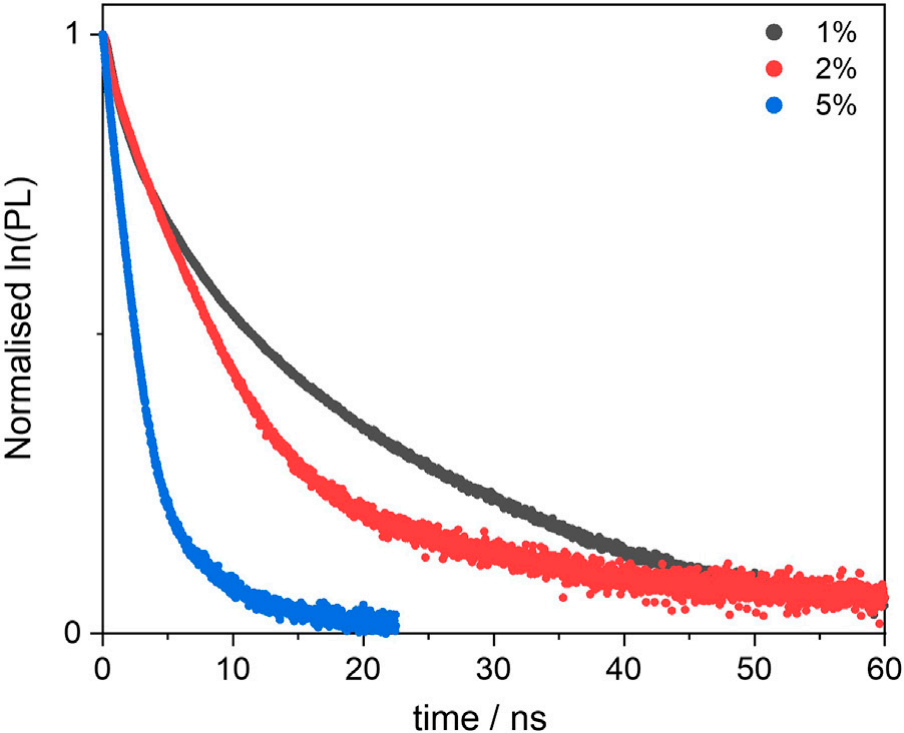
Excited state decay profiles of the BOPHY1-PMO samples, recorded at λ_em_ = 540 nm, using a 472 nm laser excitation.

We then studied the deactivation dynamics occurring at the excited states via time-resolved fluorescence. The decay profiles are shown in Figure 6 and are all fitted with biexponential functions. The overall trend sees the average lifetime decreasing with the loading and the short trace component is in good agreement with the decay of the free dye. Such trace undergoes a slight reduction to 2.3 ns in the 5% sample and this would be in line with previous findings on aggregated dyes mentioned above. It is worth mentioning also that other factors behind the lifetime decrease, besides aggregation, include the dye’s environment, which in this case is a silica matrix and might cause the formation of non-fluorescent trap sites. (Santos, 1986; Boulu et al., 1987; Méallet-Renault et al., 2006).

Interestingly, a second, long decay trace emerges in the PMO materials, about 10–12 ns, which is not found when the dye is in diluted solution. This will be discussed in a broader context in Section 3.3.2.

### 3.3 PMO materials with BOPHY2

The synthesis procedure for the BOPHY2-PMO materials was identical to that described in the previous section and, as in the previous case, three samples were prepared, with 1%, 2% and 5% dye loading (see Section 2.5).

#### 3.3.1 Structural analysis

**FIGURE 7:**
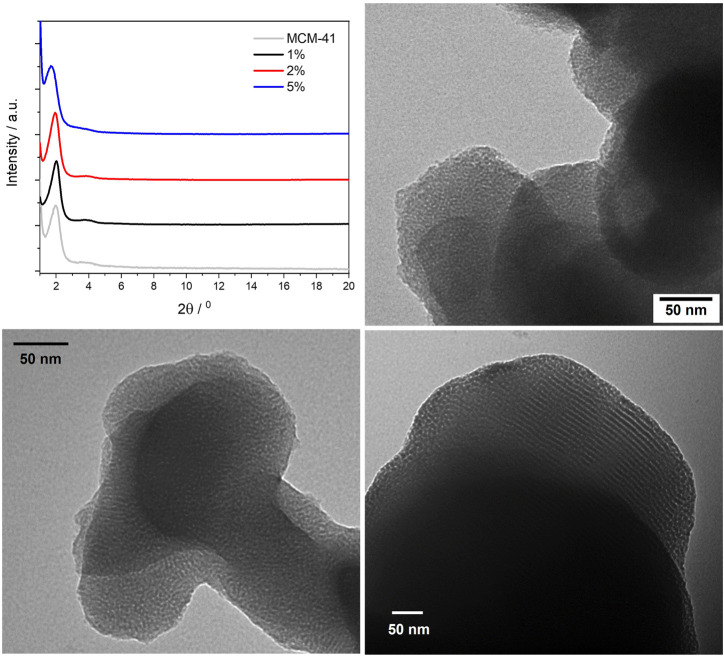
Powder X-ray diffraction patterns of the BOPHY2-PMO materials (top left); TEM images of 1% (top right), 2% (bottom left) and 5% (bottom right).

The powder XRD profiles of the three BOPHY2-PMO samples are shown in Figure 7 (top left), in comparison with plain MCM-41 silica. Monitoring the (100) reflection at 1.97° 2θ, it is possible to notice a shift towards lower angles as the dye content in the material increases. At 1% dye loading, the (100) reflection is observed at 1.97°, analogous to plain silica, at 2%, the peak shifts to 1.92°, and at 5% loading, the (100) reflection has shifted to 1.71°. This corresponds to a gradual increase in the *d*-spacing, from 4.48 to 4.60 nm, up to 5.16 nm at the highest content of dye. This is a remarkable difference in comparison with the BOPHY1-PMO materials and it must be correlated to the silica formation mechanism. The trend with the *d*-spacing can be rationalised as a consequence of the expansion of the silica network during its formation and growth around the micellar template. The introduction into the reaction mixture of an increasing quantity of bis-silylated dye, which is bulkier than TEOS, forces the growing dye-silica network to swell and wrap less tightly around the template than when only TEOS is used. It is also worth mentioning that the secondary (110) reflection is visible around 3.8° 2θ and it appears to shift slightly to lower angles in the 5% sample. This would confirm that the network expansion reflects at all dimensions to a certain extent.

The TEM images are in line with the structure and the morphology typically observed for plain silica, with particle size in the 200–300 nm range. A close inspection of the pore size, although limited in accuracy by the relatively small dimensions, reveal a gradual increase in the average diameter, from 4.2 nm in the 1% sample, to 4.7 nm in the 2% and up to 6.7 nm in the 5%.

#### 3.3.2 Photophysical properties

**FIGURE 8:**
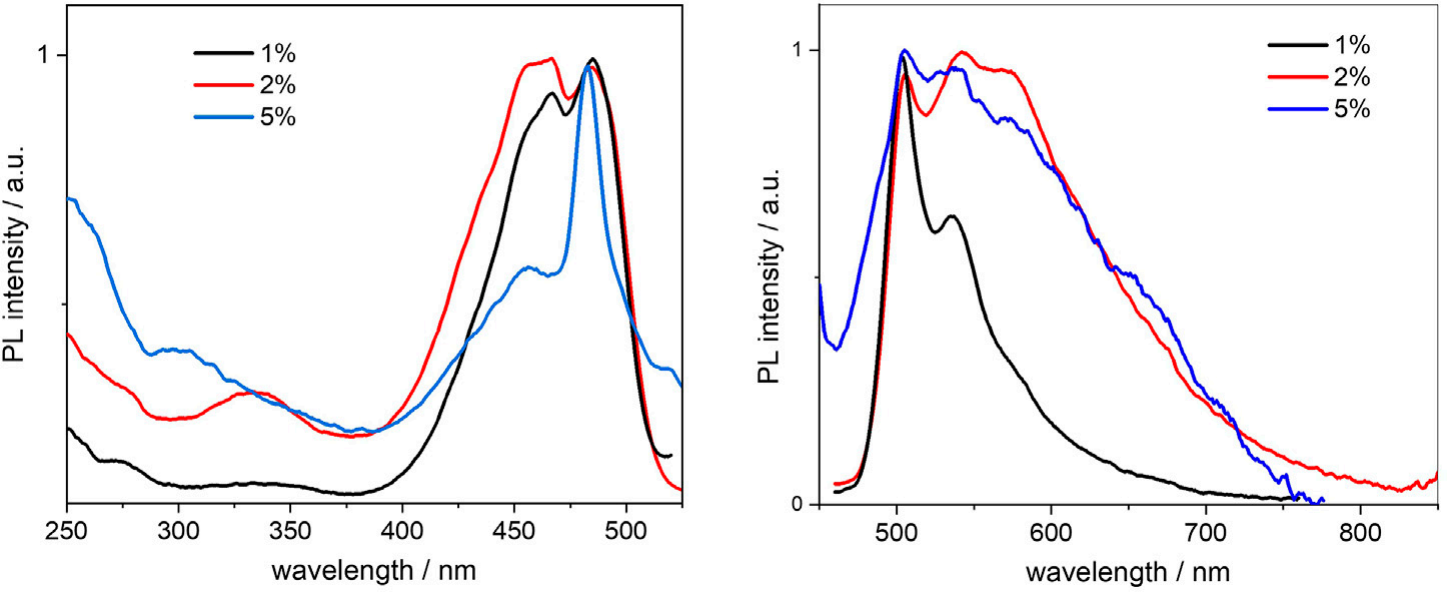
Normalised excitation (left) and emission (right) spectra of the BOPHY2-PMO samples, recorded from 0.3 mg/ml cyclohexane suspensions at λ_em_ = 540 nm and λ_exc_ = 420 nm, respectively.

The photoluminescence spectra of the BOPHY2-PMOs were recorded from diluted suspensions, as for the BOPHY1-PMOs and are shown in Figure 8. At 1% dye loading, both the excitation and the emission profiles, resemble those of the free dye, with the typical vibronic progression well visible.

Doubling the dye loading leads to a first change, as the spectra broaden, particularly the emission. This could be due to the reduction in intensity of the first and the second vibronic transitions, at 505 and 540 nm in emission, respectively. A tight close-packing between dye units within the PMO framework is likely to affect the vibrational degrees of freedom of the chromophores and will hinder some, more so those at high energy. The effect is even more pronounced at 5% loading and the vibronic progression is almost lost. It should be noted that the sharp, symmetrical peak at 456 nm (blue line in Figure 8, left side) may well be due to light scattering from the particles and it masks the 0–0 excitation transition.

**FIGURE 9:**
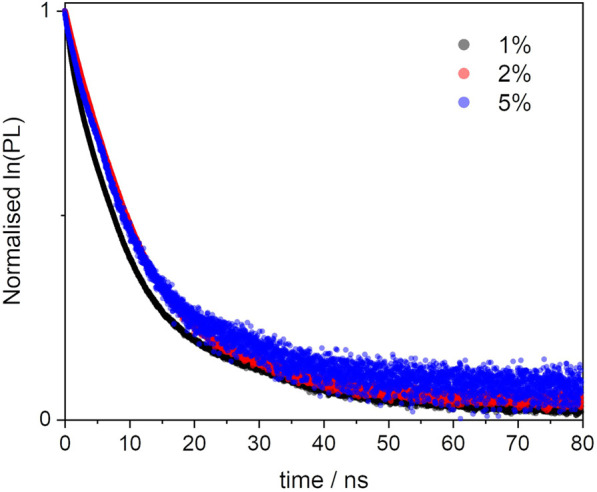
Excited state decay profiles of the BOPHY2-PMO samples, recorded at λ_em_ = 540 nm, using a 472 nm laser excitation.

The increasing concentration of dye units, which reduces the mutual distances, should be the reason for the reduction in fluorescence quantum yields from 0.63 to 0.27 (see Table 2). As discussed in Section 3.2.2, there are several factors affecting the non-radiative decay of the dye units in the solid matrix. To the intramolecular deactivation channels, represented by the S_1_ —> S_0_ internal conversion and S_1_ —> T_1_ intersystem crossing, we should add a series of intermolecular deactivation pathways, such as thermal quenching with the siloxane network, with neighbouring dye units and also via aggregates and trap sites. An interesting further trend is observed from the time-resolved measurements, so as in the BOPHY1-PMO samples. The excited-state decay lifetimes were recorded at the emission wavelength of 540 nm (Figure 9) and two decay traces were obtained, the shorter of which matches with the lifetime of the free BOPHY precursor.

**TABLE 2:** Excited-state lifetimes, fluorescence quantum yields, radiative rate constants (k_r_) and non-radiative rate constants (k_nr_) of the **BOPHY2-PMOs**.

Sample	Decay traces [ns] (relative weight)^a^	Average τ_PL_ [ns]	Φ_PL_	k_r_ [s^−1^]	k_nr_ [s^−1^]
**4**	2.8	2.8	0.86	3.1 × 10^8^	5.2 × 10^7^
**1%**	2.5 (80%)–13.0 (20%)	4.6	0.63	1.4 × 10^8^	8.0 × 10^7^
**2%**	2.7 (75%)–12.6 (25%)	4.9	0.32	6.5 × 10^7^	1.4 × 10^8^
**5%**	2.9 (48%)–14.0 (52%)	8.6	0.27	3.1 × 10^7^	8.5 × 10^7^

a Recorded at 540 nm, using a 375 nm laser excitation.

The second component is longer than 12 ns and could hardly be justified as prompt fluorescence from isolated units of dyes in the solid matrix. Excluding any impurities after repeated synthesis and measurements in water suspensions, it was then hypothesised that a form of delayed fluorescence may originate within the hybrid dye-silica structure. Since the structure is not perfectly crystalline, dye units will be found in distinct local environments. Isolated chromophores would then emit with the characteristic fluorescence lifetimes, whereas BOPHY units in close proximity with one another could show a different decay kinetics. The latter population is expected to increase with the dye loading and this is confirmed by the large relative weight of the long decay trace in the 5% sample. Within this environment, charge-transfer between adjacent units can be favoured by some forms of dipole-dipole alignment, enabling a deactivation route that BOPHY dyes do not normally show intramolecularly, despite their relatively high transition dipole moments. (Woodford et al., 2018; Hupfer et al., 2021).

In addition to this, it is possible to observe the emergence of a low-energy shoulder band in the 5% sample, both in excitation, around 517 nm, and in emission, at 650 nm. To investigate this further, an excitation spectrum at λ_em_ = 650 nm was recorded, together with an excited state lifetime at the same wavelength (see Supplementary Material S32, S33). The excitation profile does not provide conclusive evidence of a clear peak at long wavelengths and only few weak side bands can be noted up to 570 nm. Whether they could be a sign of ground-state aggregates or an effect of scattering, it is difficult to state. The fluorescence lifetime of the 5% sample, though, gives an indication of a short decay at 650 nm. Along with the traces shown in Table 2, a third component was detected, which fits with a 0.8 ns lifetime. This can be tentatively attributed to an aggregated species, most likely a ground-state aggregate of J-type. Such species typically show red-shifted spectral features compared to the monomers and their formation is favoured in linear conjugated molecules. (Takeda et al., 2016).

### 3.4 Fluorescence anisotropy studies

**FIGURE 10:**
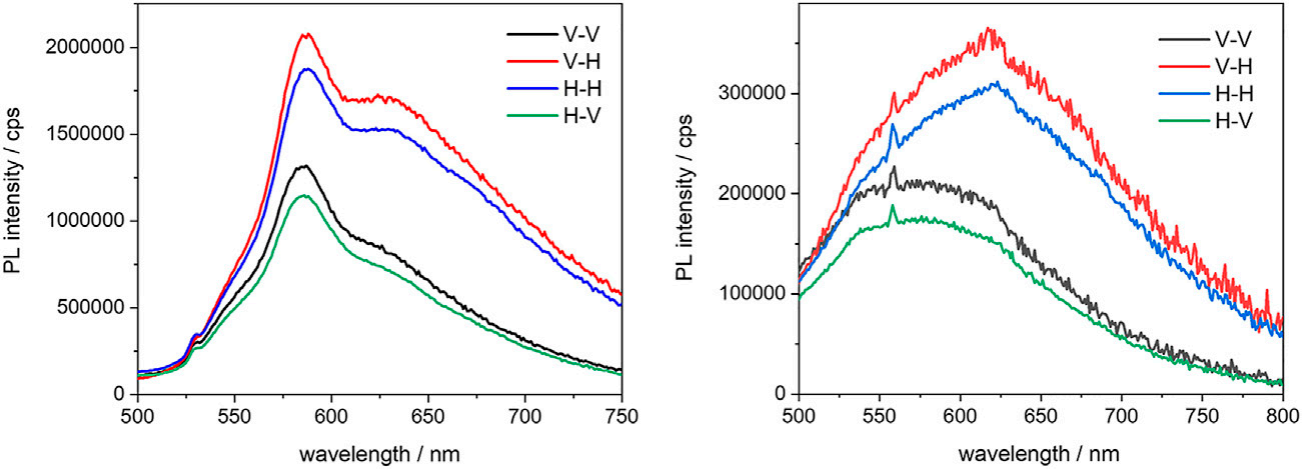
Emission anisotropy spectra of the 1% (left) and the 5% (right) BOPHY2-PMO, recorded at λ_exc_ = 470 nm from powder thin films; the notation in the legend indicates the plane-polarisation of the excitation and the emission light beam, respectively.

It was precisely because the chromophores in question have a conjugated linear structure, along which the dipole moment is oriented, that it was decided to investigate the PMOs further, using fluorescence anisotropy. All BOPHY-PMO samples were then analysed in the solid state, from thin powder films, as measurements in diluted suspensions would be affected by the continuous Brownian motion of the particles, which renders the observation of anisotropy effects extremely difficult. As the starting point, the emission and excitation spectra at different orientations of polarised light (vertical and horizontal) were first measured, to determine the G-factor and evaluate the anisotropic photoresponse of the molecules in the PMOs. All three BOPHY2-PMOs showed such a response and measurable kinetic traces, whereas among the BOPHY1-PMOs only the 1% yielded some evidence of polarisation transfer. Some samples also show different spectral features depending on the orientation of the emission; the **1%** and **5%** BOPHY2-PMO are shown in Figure 10 as the most representative, while the spectra from the other samples can be found in the Supplementary Material S34, S36.

A significant fraction of dye units seems to adopt a preferred orientation, especially in the BOPHY2-PMOs, where the chromophores are an integral part of the framework and are arranged more tightly than in the BOPHY1-PMOs, where the dyes are still part of the silica material but randomly oriented all over the PMO inner and outer surface. Nevertheless, it was possible to detect anisotropic emission for one of such samples, the **1%** BOPHY1-PMO, and a polarisation transfer trace, of about 812 ps (see Supplementary Material S34, S37).

**FIGURE 11:**
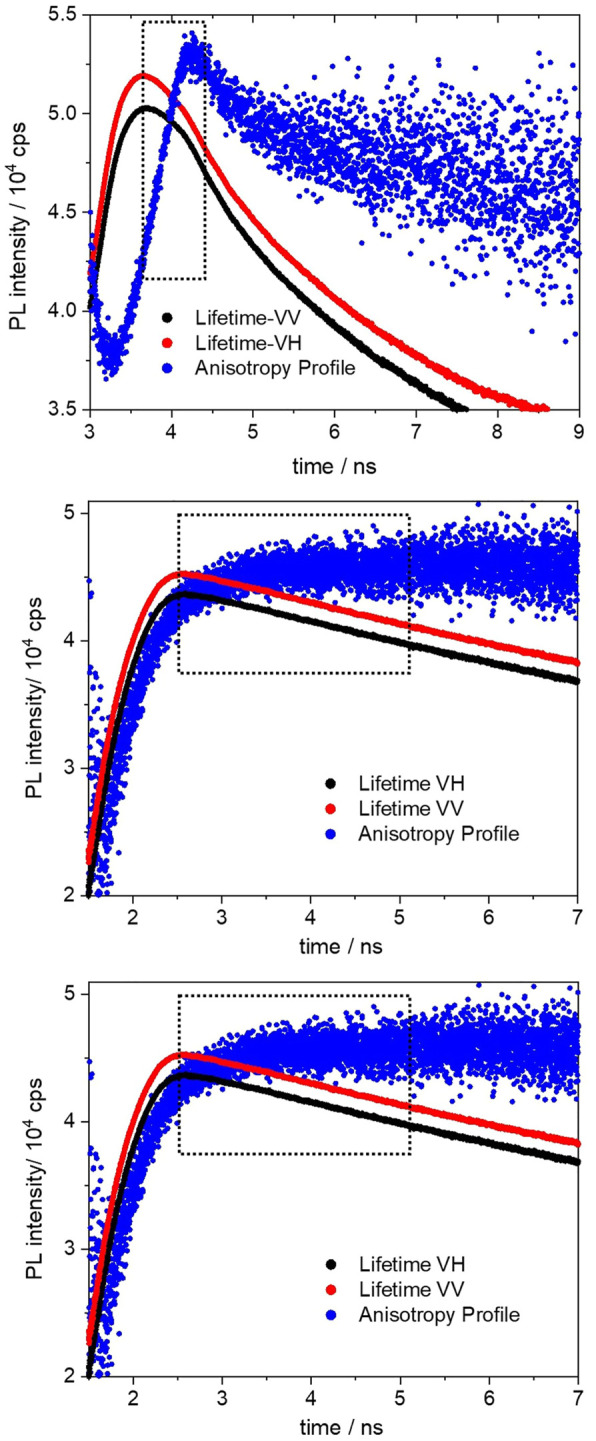
Fluorescence anisotropy decay profiles (black and red) of the 1% (top), 2% (middle) and 5% (bottom) BOPHY2-PMO, recorded at 560 nm. The blue profiles are the anisotropy curves generated from the respective decays.

The excited-state anisotropy decays of the BOPHY2-PMOs are shown in Figure 11.

The polarisation transfer time in the **1%** BOPHY2-PMO sample is the longest (783 ps) in the series. As the dye loading increases, the transfer time decreases, to 448 ps in the 2% and to 395 ps in the 5% sample. This can be ascribed as an effect of the decreasing distance between units of BOPHY dyes within the PMO framework.

The above time traces are interpreted as the times of excitation energy migration between neighbouring BOPHY units. As the excitation energy is transferred across the material, the emitted light gradually changes its orientation in comparison with the original excitation direction and the recorded kinetic profile accounts for the overall timescale of such process. (Berberan ‐ Santos et al., 1995; López Arbeloa et al., 2007; Traub et al., 2011).

There are several mechanisms that could be taken into consideration to explain how energy migration may occur in the PMOs. Pure organic materials typically display singlet-exciton diffusion and Förster energy transfer. Anisotropy studies in thin films of semiconductive polymers and aggregates of small molecules have shown evidence of multi-chain or multi-molecule energy transfer, whereby excitons are found to travel over 6 nm between excitation and emission, a lower bound for actual exciton diffusion lengths. (Traub et al., 2011). For small molecules, indeed, diffusion lengths can reach up to 65 nm, with values being sensitive to the degree of intermolecular packing in the solid state and varying greatly in crystalline and amorphous phases. (Bjorgaard and Köse, 2015). In a crude model, a hybrid organosilica material could be regarded as a nearly amorphous phase, with siloxane bridges as insulating spacers between single dye units and small dye populations where chromophores can be found in close proximity. All such distinct local environments will exhibit potentially different transfer rates, coming as a result of multiple and competing migration mechanisms.

## 4 Conclusion

The synthesis of new PMO materials containing a novel BOPHY dye was successfully achieved. Two different derivatives were synthesised from a common precursor, one bearing an alkoxysilane group and one bearing two of them, and they were incorporated separately within the walls of mesoporous silica at relatively low concentrations, up to 5% molar ratio vs TEOS.

The structure and the morphology of the PMOs was analysed using powder XRD and TEM, which proved that they are ordered materials, featuring the hexagonal pore arrangement and pore size of typical MCM-41 silica. The optical study shows that the most fluorescent materials are those with 1% dye loading, whereas increasing the content of BOPHY units in the solid matrix seem to favour non-radiative deactivation pathways. The fluorescence anisotropy experiments revealed that all materials show polarised light emission to a significant extent and, for most samples, it was also possible to determine a polarisation transfer time. This appears as a short kinetic trace and decreases from around 800 to 400 ps as the dye loading increases. This can be attributed to the decreasing distances between single units of BOPHY dyes within the PMO structure, which causes the excitation energy to migrate at an increasingly faster rate. Such property makes the new PMOs very interesting systems for further use as solid-state light-harvesters and sensitisers, although their fluorescence yields at high dye content would require further optimisation. Moreover, the emergence of a form of delayed fluorescence in the materials is opening the way to further studies on a charge-recombination mechanism and on the possibility to apply the materials in light-emitting devices.

## Data Availability

The original contributions presented in the study are included in the article/Supplementary Material, further inquiries can be directed to the corresponding author.
