# Supplementary material for: Hybridising inorganic materials with fluorescent BOPHY dyes: A structural and optical comparative study
Source: Front Chem. 2022 Jun 28;10:921112. doi: 10.3389/fchem.2022.921112 (PMC9274302; doi:10.3389/fchem.2022.921112)
Supplement: Supplementary file 1 [file DataSheet1.docx]

Supplementary Material

# Supplementary Synthesis Information

**S.1** Synthesis of (1*E*,2*E*)-1,2-Bis((3,5-dimethyl-1*H*-pyrrol-2-yl)methylene)hydrazine (**1**).

Following the method reported by Ziegler *et al*,^S1^ hydrazine hydrate (0.24 mL, 4.91 mmol, 1.00 eq) and 3,5-dimethylpyrrole-2-carboxaldehyde (1.00 g, 8.05 mmol, 1.65 eq) were dissolved in EtOH (50 mL) and a few drops of acetic acid were added. After few seconds, the solution became yellow and a precipitate formed. The reaction mixture was left to stir at room temperature for 3 h. The precipitate was collected via filtration, washed with cold EtOH (20 mL), and dried under vacuum to give the title compound **1** as a yellow powder (1.70 g, 86%), mp 241-244 °C (lit.^S2^ 236-240 ºC). R*_f_* = 0.2 (petroleum ether: CH_2_Cl_2_ 1:1). ^1^H NMR (300 MHz, CDCl_3_) δ 8.76 (br s, 2H), 8.37 (s, 2H), 5.81 (s, 2H), 2.27 (s, 6H), 2.18 (s, 6H).^13^C NMR (75 MHz, CDCl_3_) δ 147.57, 133.14, 127.34, 123.26, 110.68, 13.17, 10.72. IR (neat): ν_max_/cm^-1^: 3450, 2960, 1615, 1552, 1446, 1375, 1256, 1158, 1050, 991, 870, 806, 686, 540.

The data obtained for this compound is consistent with the literature.^S2^

**S.2** ^1^H NMR spectrum of **1** (300 MHz, CDCl_3_).

**S.3** ^13^C NMR spectrum of **1** (75 MHz, CDCl_3_).

**S.4** Synthesis of 5,5,12,12-tetrafluoro-1,3,8,10-tetramethyl-5*H*,12*H*-5l4,6l4,12l4,13l4-pyrrolo[1,2-d]pyrrolo[1',2':4,5][1,2,4,3]triazaborinino[2,1-a][1,2,4,3]triazaborinine (**2**).

Adapted from the method reported by Son,^S2^ (1*E*,2*E*)-1,2-Bis((3,5-dimethyl-1*H*-pyrrol-2-yl)methylene)hydrazine **1** (0.80 g, 3.30 mmol, 1 eq) was dissolved in toluene (25 mL) and the solution was stirred at room temperature for 30 min. *N,N*-Diisopropylethylamine (6.5 g, 50 mmol, 8.7 mL, 15 eq) and BF_3_.Et_2_O (10.0 g, 8.7 mL, 71 mmol, 21 eq) were then added dropwise and the reaction mixture was heated under reflux for 20 h. The reaction mixture was allowed to cool to room temperature, quenched with cold water (20 mL) and extracted with CH_2_Cl_2_ (2 × 30 mL). The combined organic layers were dried over MgSO_4_ and filtered. The organic solvent was removed under reduced pressure to yield a solid which was purified by column chromatography (petroleum ether:CH_2_Cl_2_ 1:1) to give the title compound **2** as a dark brown solid (0.70 g, 64%), mp 248-252 °C (lit.^S2^ 250-253 °C). R*_f_* = 0.32 (petroleum ether:CH_2_Cl_2_ 1:1). ^1^H NMR (300 MHz, CDCl_3_) δ 7.94 (s, 2H), 6.18 (s, 2H), 2.49 (s, 6H), 2.33 (s, 6H). ^13^C NMR (75 MHz, CDCl_3_) δ 151.21, 141.03, 134.56, 123.39, 118.54, 14.12, 11.10. ^11^B NMR (96 MHz, CDCl_3_) δ 0.70 (t, *J_B-F_* = 28.1 Hz). ^19^F NMR (282 MHz, CDCl_3_) δ -142.36 (q, *J_F-B_* = 28.5 Hz). IR (neat): ν_max_/cm^-1^: 1589, 1520, 1307, 1225, 1135, 1085, 1042, 960, 929, 843, 804, 722, 639, 585.

The data obtained for this compound is consistent with the literature.^S2^

**S.5** ^1^H NMR spectrum of **2** (300 MHz, CDCl_3_).

**S.6** ^13^C NMR spectrum of **2** (75 MHz, CDCl_3_).

**S.7** ^11^B NMR spectrum of **2** (96 MHz, CDCl_3_).

**S.8** ^19^F NMR spectrum of **2** (282 MHz, CDCl_3_).

**S.9** Synthesis of 5,5,12,12-tetrafluoro-2,9-diiodo-1,3,8,10-tetramethyl-5*H*,12*H*-5l4,6l4,12l4,13l4-pyrrolo[1,2-d]pyrrolo[1',2':4,5][1,2,4,3]triazaborinino[2,1-a][1,2,4,3]triazaborinine (**3**).

Following the method reported by Ziessel,^S3^ 5,5,12,12-tetrafluoro-1,3,8,10-tetramethyl-5*H*,12*H*-5l4,6l4,12l4,13l4-pyrrolo[1,2-d]pyrrolo[1',2':4,5][1,2,4,3]triazaborinino[2,1-a][1,2,4,3]triazaborinine **2** (0.500 g, 1.48 mmol, 1 eq) was placed in flame-dried Schlenk tube and diluted with chloroform (60 mL). Iodine monochloride (2.69 g, 16.6 mmol, 11.2 eq) in methanol (20 mL) was added dropwise to the reaction mixture. After few minutes, a precipitate formed and the reaction mixture was stirred at room temperature for 3.5 h. After that, the reaction mixture was washed with a saturated aqueous solution of Na_2_S_2_O_3_ (2 × 15 mL) and water (2 × 15 mL). The combined organic layers were dried over MgSO_4_ and filtered. The organic solvent was removed under reduced pressure to give the title compound **3** as a light red solid (0.61 g, 70%), mp 209-213 °C (lit^S2^ 205-208 °C), R*_f_*  = 0.6 (petroleum ether:CH_2_Cl_2_ 1:1). ^1^H NMR (300 MHz, CDCl_3_) δ 7.99 (s, 2H), 2.55 (s, 6H), 2.31 (s, 6H). ^13^C NMR (75 MHz, CDCl_3_) δ 152.19, 143.56, 135.09, 123.33, 80.97, 15.17, 13.61. ^11^B NMR (96 MHz, CDCl_3_) δ 0.49 (t, *J*_B-F_ = 28.5 Hz). ^19^F NMR (282 MHz, CDCl_3_) δ -142.12 (q, *J_F-B_* = 27.5 Hz). ν_max_/cm^-1^: 1594, 1455, 1384, 1310, 1214, 1154, 1107, 1056, 983, 942, 908, 858, 742, 599.

The data obtained for this compound is consistent with the literature.^S2^

**S.10** ^1^H NMR spectrum of **3** (300 MHz, CDCl_3_).

**S.11** ^13^C NMR spectrum of **3** (75 MHz, CDCl_3_).

**S.12** ^11^B NMR spectrum of **3** (96 MHz, CDCl_3_).

**S.13** ^19^F NMR spectrum of **3** (282 MHz, CDCl_3_).

**S.14** ^1^H NMR spectrum of **4** (300 MHz, CDCl_3_).

**S.15** ^13^C NMR spectrum of **4** (101 MHz, CDCl_3_).

**S.16** ^11^B NMR spectrum of **4** (96 MHz, CDCl_3_).

**S.17** ^19^F NMR spectrum of **4** (282 MHz, CDCl_3_).

**S.18** ^1^H NMR spectrum of **BOPHY1** (300 MHz, CDCl_3_).

**S.19** ^13^C NMR spectrum of **BOPHY1** (101 MHz, CDCl_3_).

**S.20** ^19^F NMR spectrum of **BOPHY1** (282 MHz, CDCl_3_).

**S.21** ^11^B NMR spectrum of **BOPHY1** (96 MHz, CDCl_3_).

**S.22** ^1^H NMR spectrum of **BOPHY2** (300 MHz, CDCl_3_).

**S.23** ^13^C NMR spectrum of **BOPHY2** (101 MHz, CDCl_3_).

**S.24** ^19^F NMR spectrum of **BOPHY2** (282 MHz, CDCl_3_).

**S.25** ^11^B NMR spectrum of **BOPHY2** (128 MHz, CDCl_3_).

# Supplementary UV-visible Spectroscopy Data

**S.26** Beer-Lambert plot for **4**, with absorbance recorded at λ = 460 nm in THF, plotted against molar concentration of a 5-point dilution series.

**S.27** Excited state decay of the BOPHY dye **4**, recorded at 540 nm from a 10^-6^ M THF solution, using a 472 nm laser excitation.

**S.28** Excitation spectra of the **BOPHY1-PMO** materials, recorded from 0.3 mg/mL cyclohexane suspensions at λ_em_ = 540 nm.

**S.29** Emission spectra of the **BOPHY1-PMO** materials, recorded from 0.3 mg/mL cyclohexane suspensions at λ_exc_ = 420 nm.

**S.30** Excitation spectra of the **BOPHY2-PMO** materials, recorded from 0.3 mg/mL cyclohexane suspensions at λ_em_ = 540 nm.

**S.31** Emission spectra of the **BOPHY2-PMO** materials, recorded from 0.3 mg/mL cyclohexane suspensions at λ_exc_ = 420 nm.

**S.32** Emission spectra of the **BOPHY2-PMO** material at 5% dye loading, recorded from a 0.3 mg/mL cyclohexane suspension at λ_em_ = 620 nm.

**S.33** Excited state decay profiles of the **BOPHY2-PMO** with 5% dye loading, recorded at λ_em_ = 540 nm and at 650 nm, using a 472 nm laser excitation.

**S.34** Emission (left) and excitation (right) anisotropy spectra of the **1% BOPHY1-PMO**, recorded at λ_exc_ = 480 nm and λ_em_ = 560 nm, respectively, from a powder thin film; the notation in the legend indicates the plane-polarisation of the excitation and the emission light beam. G-factor = 1.81 at 560 nm.


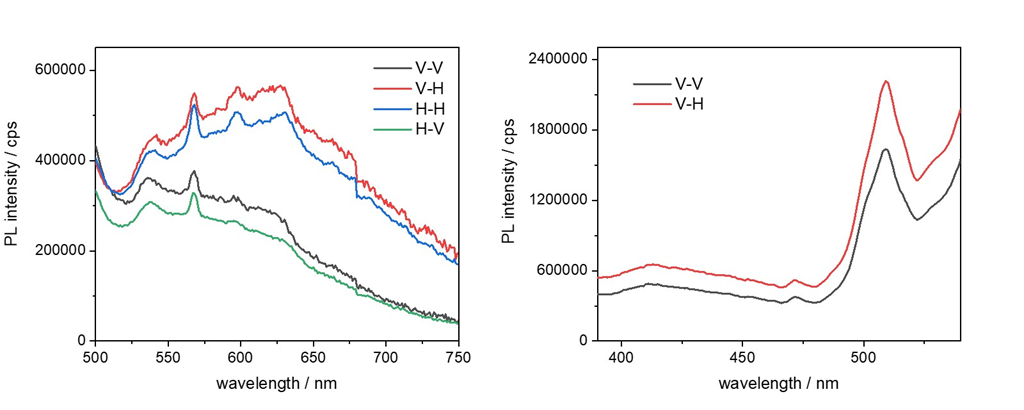


**S.35** Excitation anisotropy spectra of the **1%** (left) and the **5%** (right) **BOPHY2-PMO**, recorded at λ_em_ = 620 nm, from powder thin films; the notation in the legend indicates the plane-polarisation of the excitation and the emission light beam. G-factor at 620 nm: 2.41 at 1% and 2.45 at 5%.

**S.36** Emission (left) and excitation (right) anisotropy spectra of the **2% BOPHY2-PMO**, recorded at λ_exc_ = 470 nm and λ_em_ = 620 nm, respectively, from a powder thin film; the notation in the legend indicates the plane-polarisation of the excitation and the emission light beam. G-factor = 2.45 at 620 nm.


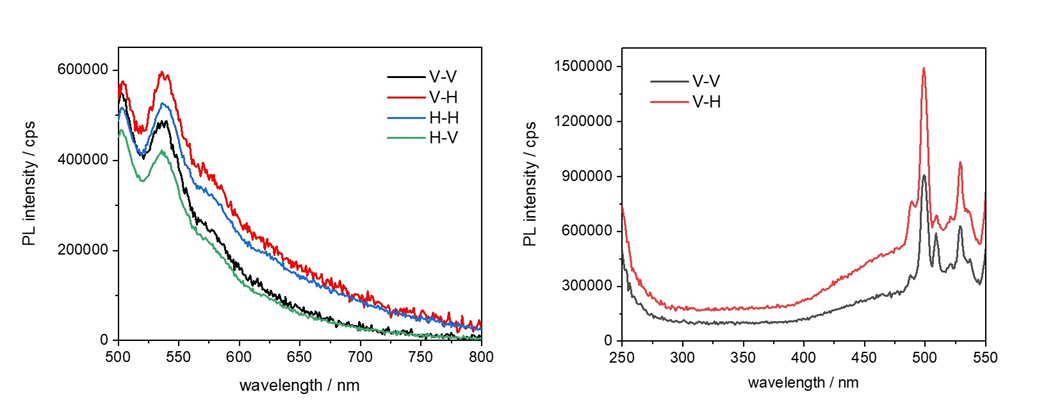


**S.37** Top: Fluorescence anisotropy decay profiles (black and red) of the **1% BOPHY1-PMO**, recorded at 560 nm. The blue profile is the anisotropy curve generated from the two decays. Bottom: fitted time trace from the anisotropy profile, obtained in the range indicated by the dotted square in the top part.


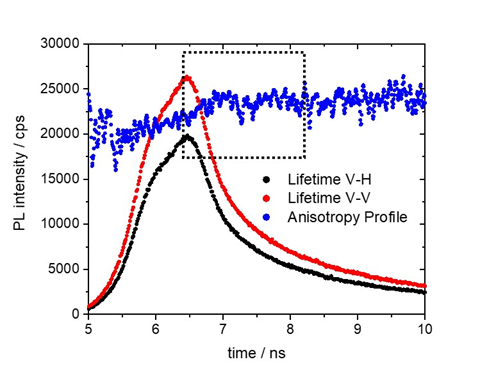

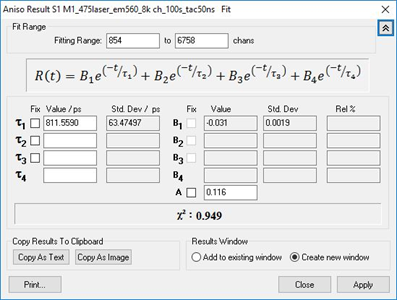


**S.38** Fitted time traces from the anisotropy profiles shown in **Figure 14**, obtained in the ranges indicated by the dotted squares. Top: **1% BOPHY2-PMO**; middle: **2% BOPHY2-PMO**; bottom: **5% BOPHY2-PMO**.


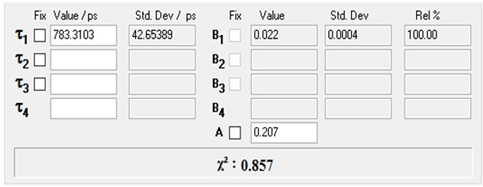


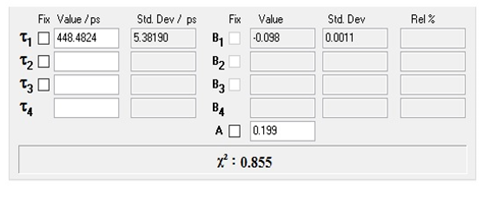


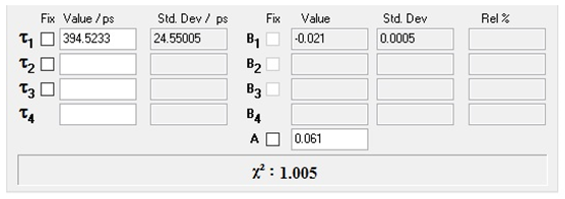


**References**

(S1) I. S. Tamgho, A. Hasheminasab, J. T. Engle, V. N. Nemykin, C. J. Ziegler, *J. Am. Chem. Soc.* **2014**, *136*, 5623-5626.

(S2) X. Li, G. Ji, Y.-A. Son, *Dyes and Pigments* **2016**, *124*, 232-240.

(S3) Q. Huaulmé, A. Mirloup, P. Retailleau, R. Ziessel, *Org. Lett.* **2015**, *17*, 2246-2249.
